# Striatal direct and indirect pathways control decision-making behavior

**DOI:** 10.3389/fpsyg.2014.01301

**Published:** 2014-11-12

**Authors:** Tom Macpherson, Makiko Morita, Takatoshi Hikida

**Affiliations:** Medical Innovation Center, Kyoto University Graduate School of MedicineKyoto, Japan

**Keywords:** nucleus accumbens, dorsal striatum, medium spiny neurons, direct striatal pathway, indirect striatal pathway, social behavior, goal-directed behavior, action selection

## Abstract

Despite our ever-changing environment, animals are remarkably adept at selecting courses of action that are predictive of optimal outcomes. While requiring the contribution of a number of brain regions, a vast body of evidence implicates striatal mechanisms of associative learning and action selection to be critical to this ability. While numerous models of striatal-based decision-making have been developed, it is only recently that we have begun to understand the precise contributions of specific subpopulations of striatal neurons. Studies utilizing contemporary cell-type-specific technologies indicate that striatal output pathways play distinct roles in controlling goal-directed and social behaviors. Here we review current models of striatal-based decision-making, discuss recent developments in defining the functional roles of striatal output pathways, and assess how striatal dysfunction may contribute to the etiology of various neuropathologies.

## INTRODUCTION

Economical decision-making can be defined as the selection of the optimal (i.e., most rewarding or least aversive) course of action among a host of competing alternatives. This process requires a system that is capable of: (1) encoding associations between actions and the predicted value of their outcomes, (2) initiating selected actions while suppressing competing non-selected actions, and (3) dynamically adapting behavior in response to changes in outcome value. Although complex decision-making is thought to rely on a widely distributed neural network, including cortical, limbic and midbrain regions, efferent projections from these structures are known to converge within the striatum of the basal ganglia (Alexander and Crutcher, 1990; Haber, 2003). Indeed, the striatum is hypothesized to integrate cognitive, emotional, and motivational information that help to guide to the selection of economical actions (Mogenson et al., 1980). This review will begin by summarizing the role of striatal mechanisms in neuropsychological processes associated with economical decision-making. Subsequently, we will discuss how new technologies have begun to elucidate discrete roles for striatal cell-types and output pathways, and how these may contribute to decision-making-associated neuropathologies. Finally, we will review recent evidence that economical decision-making guiding individual and social behavior may share common molecular mechanisms and neurocircuitry.

## ANATOMY OF THE STRIATUM

The striatum, the largest component and primary afferent structure of the basal ganglia, is anatomically linked to the cerebral cortex, limbic system and thalamo-cortical motor system via a series of parallel, but largely structurally and functionally distinct cortico-subcortical circuits (Holland and Rescorla, 1975; Gerfen and Young, 1988; Alexander and Crutcher, 1990; Dickinson and Balleine, 1994; Haber, 2003). The dorsomedial and dorsolateral regions of the striatum receive afferent projections from frontal and parietal associated cortices, and sensorimotor cortices, respectively. Whereas, the ventral striatum, largely comprised of the nucleus accumbens (NAc), receives projections from limbic structures, including the amygdala, hippocampus as well as the medial prefrontal and anterior cingulate cortices (Alexander et al., 1986; Haber, 2003). This topography is proposed to confer dissociable functions to each of the striatal subregions, allowing them to dynamically and adaptively control the flow of cognitive and affective information to motor output systems, resulting in facilitation or inhibition of actions (Mink, 1996; Balleine and Dickinson, 1998; Nicola, 2006).

## STRIATAL-MEDIATED LEARNING

The striatum acts to support selection of economical actions through its role in mediating two different forms of associative learning (Balleine et al., 2007; Liljeholm and O’Doherty, 2012). The first, Pavlovian (stimulus-response) learning, describes the process by which an initially neutral conditioned stimulus (CS), by repeated pairing with an unconditioned stimulus (US) eliciting an unconditioned response (UR), acquires the capacity to evoke the same, now conditioned, response (CR). Whereas, in instrumental (action-outcome) learning, the likelihood of performance of a specific behavior is modified by the appetitive or aversive outcome (US) it is associated with. While stimulus-response and the early stages of action-outcome contingencies are sensitive to devaluation of the US, following repeated training action-outcome associations become habitual, regardless of changes to the outcome (Holland and Rescorla, 1975; Dickinson and Balleine, 1994; Balleine and Dickinson, 1998). These associative learning strategies act to facilitate the likelihood of incurring economic outcomes predicted by conditioned stimuli or actions, while also conserving energy expended during cognitive processing. Interestingly, stimulus-response and action-outcome associations are not mutually exclusive and often interact with each other, such as in Pavlovian-to-instrumental transfer (PIT), in which instrumental responding for a US is facilitated by presentation of a CS that was previously paired with the same US (specific PIT) or a different US (general PIT).

**FIGURE 1:**
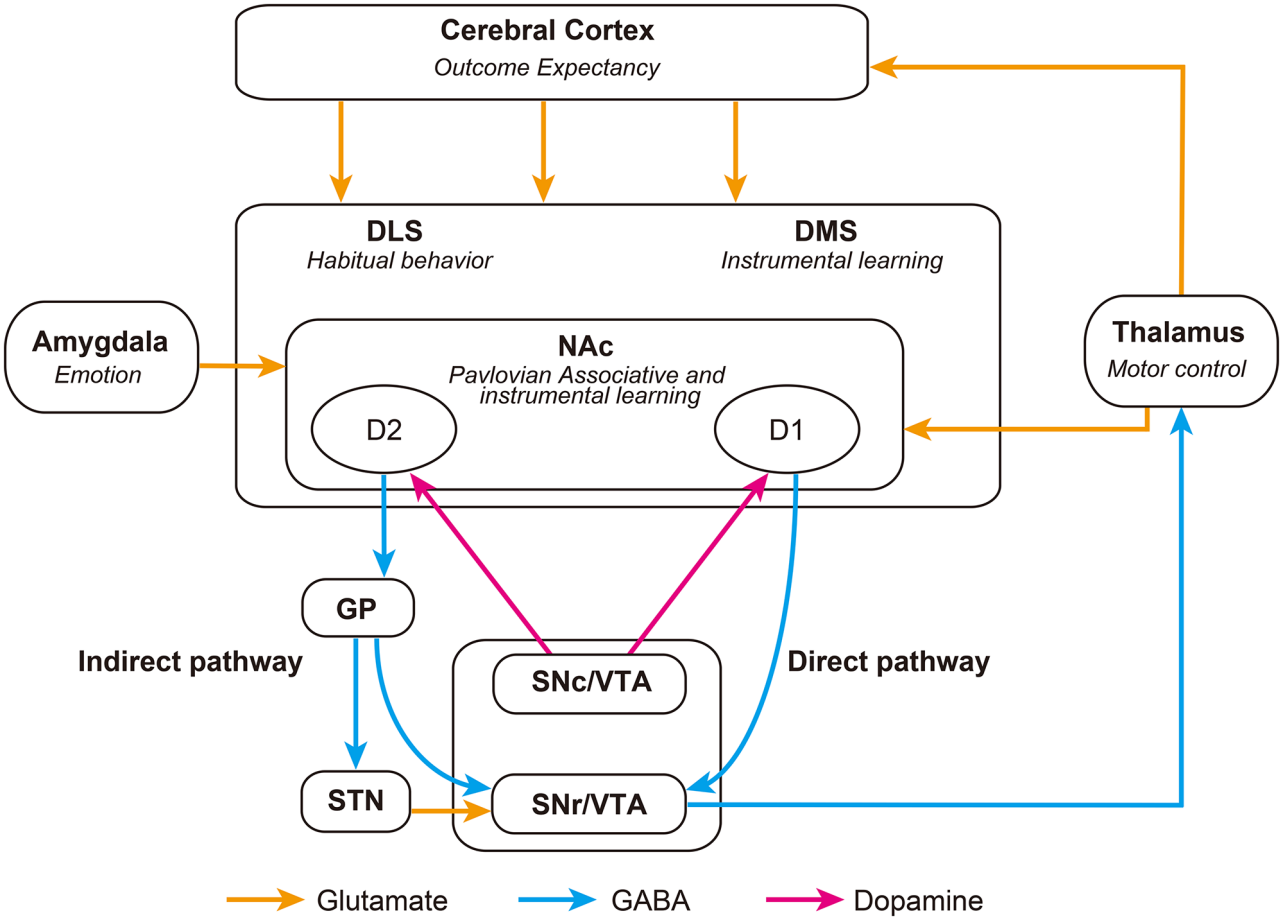
**Schematic representation of striatal-associated decision-making neurocircuitry.** Striatonigral D1 *direct* pathway neurons inhibit the SNr and release inhibition of thalamic activity, promoting behavior. Whereas, striatonigral D2 *indirect* pathway neurons inhibit the GP, disinhibiting the STN and exciting the SNr, which ultimately inhibits the thalamus and thus suppresses behavior. The balance between these opposing projections is likely to be regulated by glutamatergic and dopaminergic afferents, as well as GABAergic signaling within the striatum. DLS, Dorsolateral Striatum; DMS, Dorsomedial Striatum; GP, Globus Pallidus; NAc, Nucleus Accumbens; SNc, Substancia Nigra pars compacta; SNr, Substancia Nigra pars reticulata; STN, Subthalamic Nucleus; VTA, Ventral Tegmental Area.

As well as being delineated by their various afferents, subregions of the striatum are also functionally dissociable (**Figure 1**). The NAc of the ventral striatum, specifically the core region and its inputs from the basolateral amygdala (BLA), are implicated in the mediation of Pavlovian and instrumental conditioning [reviewed in (Everitt et al., 2001; Cardinal and Everitt, 2004)]. Furthermore, specific ‘hotspots’ within the NAc shell have been shown to mediate hedonic reactions or ‘liking’ for food and drug reward (Pecina and Berridge, 2005; Castro and Berridge, 2014). In contrast, the dorsal striatum is implicated in the control of instrumental behavior by stimulus-action associations [reviewed in (Robbins, 2002; Balleine et al., 2007)]. Interestingly, lesions to the dorsomedial striatum (DMS) inhibit goal-directed instrumental conditioning, while lesions to the dorsolateral striatum (DLS) disrupt habit formation, indicating that the DMS and DLS mediate the initial acquisition and later consolidation phases of skill learning, respectively (Yin et al., 2004, 2005, 2009). Indeed, the switch from voluntary to habitual and compulsive drug use in addiction is hypothesized to represent a neural transition in the control of behavior from ventral to dorsal striatal regions (Everitt and Robbins, 2005).

## STRIATAL MECHANISMS CONTROLLING SELECTION OF ECONOMICAL ACTIONS

While the neural mechanisms by which action selection occurs are still largely unclear, a compelling hypothesis posits that neuronal ensembles within the striatum may encode specific action representations, which when selected act to disinhibit downstream motor output nuclei (Mink, 1996; Gurney et al., 2001). In this model, actions are selected by ‘signals’ provided by input channels from cortical and limbic regions, with the most salient signal (or strongest input) winning over behavioral control. However, this model fails to explain how striatal-mediated learning influences action selection. More recently, updated models have suggested that phasic bursts of dopamine and their resulting plasticity may act to discriminatively amplify neuronal ensembles within the striatum, effectively reducing the signal-to-noise ratio (Frank, 2005; Frank and Claus, 2006; Nicola, 2006; Schroll and Hamker, 2013). This proposal is greatly influenced by the work of Schultz et al. (1997) indicating that phasic bursts of dopamine within the striatum facilitate reward-related learning by signaling reward-prediction errors (Schultz, 1998). Moreover, these models begin to implicate specific striatal cell types in the control of behavior.

Medium spiny neurons (MSNs), the primary cell type within the striatum, are typically divided into two subpopulations based upon their expression of dopamine receptors, releasable peptides and their axonal projection targets (Gerfen and Young, 1988). Dopamine D1-receptor, dynorphin-, and substance P-expressing striatonigral neurons, and D2-receptor and enkephalin-expressing striatopallidal neurons, form integral parts of the *direct* and *indirect* striatal output pathways, respectively (**Figure 1**; Gerfen et al., 1990; Surmeier et al., 1996; Bertran-Gonzalez et al., 2010). Activation of dopamine D1-receptors by phasic bursts of dopamine induces long-term potentiation (LTP) of glutamatergic synapses on striatonigral MSNs, facilitating signaling through the *direct* pathway (Grace et al., 2007; Gerfen and Surmeier, 2011). While activation of D2-receptors induces long-term depression (LTD) in striatopallidal MSNs, producing a blockade of the *indirect* pathway (Kreitzer and Malenka, 2007; Shen et al., 2008). Thus altered availability of dopamine in the striatum, induced by presentation of conditioned stimuli, is able to dynamically alter activity in *direct* and *indirect* striatal output pathways. The implications of this will be discussed in the next section.

Action selection may be additionally facilitated by GABAergic lateral inhibition of competing interactions between NAc single projection neurons (Nicola and Deadwyler, 2000; Taverna et al., 2004, 2005). Interestingly, recent evidence indicates dopamine to increase GABAergic tonic current in striatonigral MSNs, while decreasing tonic inhibition in striatopallidal MSNs (Liang et al., 2014; Maguire et al., 2014). This newly discovered mechanism might act as a neuroprotective mechanism against maladaptive behaviors associated with prolonged activiation of NAc neurons by dopamine, as with drug-induced dopamine release (Maguire et al., 2014).

## STRIATAL PATHWAYS AND THE CONTROL OF MOVEMENT

As previously alluded to, the influence of neuronal afferents coding for specific actions or tasks are likely modulated with the support of the *direct* and *indirect* striatal output pathways (**Table 1**; Kravitz et al., 2010; Fukabori et al., 2012; Nishizawa et al., 2012; Tai et al., 2012; Freeze et al., 2013). These pathways converge within the substantia nigra pars reticulata (SNr), where they dynamically control the activity of afferents to the thalamus, and consequently produce opposing influences on motor output systems (DeLong, 1990; Deniau et al., 2007). Optical stimulation of the *direct* pathway promotes motor activity, whereas stimulation of *indirect* pathway inhibits motor activity (Kravitz et al., 2010). More recently it has been proposed that cooperative activity in both pathways may be necessary for action selection and initiation. Time-correlated single-photon counting demonstrates concurrent activation of selected *direct* and *indirect* pathway striatal neurons prior to initiation of directed movement (Cui et al., 2013). It is possible that synchronized activity of individual *direct* and *indirect* pathway neurons may act to integrate the various antagonistic spatiotemporal components needed to complete motor behaviors (Isomura et al., 2013). According to this model, increases in motor activity observed following ablation (Durieux et al., 2009) or disruption (Bateup et al., 2010) of *indirect* pathway neurons, can be explained as an inability of the *indirect* pathway to inhibit competing action representations, resulting in hyperkinesia.

**Table 1:** Effects of cell-specific genetic manipulation of *direct* and *indirect* pathway neurons within different striatal regions.

	Direct pathway	Indirect pathway
NAc	Reward-learning (Hikida et al., 2010, 2013; Lobo et al., 2010; Kravitz et al., 2012)	Aversion-learning (Hikida et al., 2010, 2013; Kravitz et al., 2012; Danjo et al., 2014)
DS (unspecified)	Increased motor behavior (Kravitz et al., 2010) Increased the value of an action contralateral to a bilateral infusion ( Tai et al., 2012)	Decreased motor behavior (Kravitz et al., 2010) Increased the value of an action ipsilateral to a bilateral infusion ( Tai et al., 2012)
DMS	Regulates correct response time in performance of visual-discrimination (Fukabori et al., 2012) Inhibits SNr neurons predicting movement (Freeze et al., 2013)	Excites SNr neurons predicting motor suppression (Freeze et al., 2013)
DLS		Regulates correct response accuracy in performance of audio discrimination (Nishizawa et al., 2012)

*NAc, Nucleus Accumbens; DS, Dorsal Striatum; DMS, Dorsomedial Striatum; DLS, Dorsolateral Striatum; SNr, Substancia Nigra pars reticulata*.

Interestingly, it has also been proposed that rather than, or in addition to, simply controlling movement, striatal output pathways act to influence behavior by the inference of value to specific actions (Samejima et al., 2005; Tai et al., 2012). Optical activation of dorsomedial striatal *direct* or *indirect* pathway neurons biased action selection for a nosepoke hole located contralateral or ipsilateral to the side of stimulation, respectively. (Tai et al., 2012). This bias mimicked an additive shift in the action value estimated by the mice’s previous behavior and reward history.

## STRIATAL PATHWAY CONTROL OF GOAL-DIRECTED BEHAVIOR

There is now considerable evidence to indicate that striatal pathways are also implicated in the control of goal-directed behavior, including the acquisition of rewarding stimuli and the avoidance of aversive stimuli (**Table 1**; Hikida et al., 2010, 2013; Lobo et al., 2010; Kravitz et al., 2012; Danjo et al., 2014). Reversible-neurotransmitter-blocking (RNB) inhibition of *direct* pathway neurons attenuated the conditioned place preference (CPP) for a chamber previously paired with a food reward in a test of food-conditioned, while inhibition of the *indirect* pathway had no effect on food-CPP (Hikida et al., 2010). Conversely, RNB disruption of *indirect* pathway neurons, but not *direct* pathway neurons, blocks passive avoidance learning (Hikida et al., 2010). Thus it appears that *direct* and *indirect* striatal pathways are critical for reward- and aversion-learning, respectively. This idea is supported by optogenetic evidence demonstrating activation of *direct* pathway neurons in the NAc to induce persistent reinforcement, while stimulation of *indirect* pathway neurons was sufficient for persistent avoidance (Kravitz et al., 2012). Subsequent investigation has revealed that the ability of the *direct* pathway to facilitate reward-based learning is contingent upon the activation of dopamine D1-receptors within the NAc, while specific inactivation of NAc D2-receptors within the *indirect* pathway underlies passive avoidance learning (Hikida et al., 2013).

In addition to dopamine receptors, several other receptor types expressed in striatal pathway neurons have been implicated in the control of goal-directed behaviors. Indeed, the sphingosine-1-phosphate receptor Gpr6 and A_2A_ receptor, expressed selectively in striatopallidal neurons, control instrumental learning, likely by influencing *indirect* pathway activity (Lobo et al., 2007; Yu et al., 2009).

Interestingly, recent evidence indicates that the striatal pathways also control retention and flexibility of reward-related learning, critical for economical action selection in the face of constant or changing outcomes. Designer receptor exclusively activated by a designer drug (DREADD) activation of direct pathway neurons in the DMS significantly enhances retention of economic strategies in a reward discrimination task (Ferguson et al., 2013). Conversely, RNB inactivation of D2-receptors within the NAc is necessary for flexibly learning a new strategy, as well as suppressing the previously learned strategy in a visual-discrimination task (Yawata et al., 2012).

## THE ROLE OF THE STRIATUM IN SOCIAL ECONOMIC DECISION-MAKING

Up until now we have described how the balance of neural activity within specific striatal subpopulations contributes to decision-making processes based upon an individual’s personal value representations and behavior. However, in many circumstances decision-making is influenced by the needs of the social groups to which an individual belongs. Furthermore, social interactions, including parental attention, mutual grooming and pair-bonding, can in themselves act as reward. Accordingly, the striatum is known to contribute significantly to the organization of social behaviors (Báez-Mendoza and Schultz, 2013).

Fast scan voltammetry in rodents demonstrates dopamine release within the NAc to correlate with instances of social interaction (Robinson et al., 2002, 2011). Similarly, dopamine transmission within the NAc rostral shell, but not caudal shell or core, facilitates pair-bond formation in prairie voles (Aragona et al., 2005). Further investigation revealed D1 and D2 receptors within the NAc rostral shell to produce opposing influences on pair-bond formation, promoting and inhibiting its development, respectively (Aragona et al., 2005). Indeed, the pair-bond formation was associated with an upregulation of D1 receptors within the NAc (Aragona et al., 2005). Interestingly, evidence indicates that the rewarding properties of social interaction additionally requires the coordinated action of oxytocin and serotonin upon both D1- and D2-MSNs of the NAc (Dölen et al., 2013).

More recently, a set of studies by Gunaydin et al. (2014) has begun to elucidate specific neural pathways underlying social behavior. Social interaction in mice, but not novel object interation, was predicted by increased activity in ventral tegmental area (VTA) dopamine neurons projecting to the NAc. Accordingly, optical activation of VTA-NAc dopamine projection neurons, enhancing phasic dopamine release within the NAc, was demonstrated to increase social interaction behavior. Interestingly, intra-NAc infusion of a dopamine D1- but not D2-receptor antagonist was able to block the prosocial effects of optical VTA stimulation, while optical activation of NAc D1-MSNs was sufficient to increase social interaction. These data provide additional evidence that social behaviors can act as natural reward that are controlled through NAc D1-MSNs within the *direct* pathway. Moreover, these studies indicate that neural circuits implicated in mediating individual and social decision-making processes share common neural circuits.

Finally, recent evidence reveals that accumbens MSNs are able to bidirectional control behavioral outcomes to social stress. Artificial activation of D1-MSNs reversed social avoidance and anhedonia behaviors induced by chronic social defeat stress in mice, while inhibition of these neurons increased depression-like behaviors (Francis et al., 2014). Conversely, enhancement of NAc D2-MSN activity induced social avoidance following subthreshold social defeat stress (Francis et al., 2014). These data suggest that NAc D1- and D2-MSNs may provide efficacious targets for the treatment of disorders associated with social avoidance and depression.

## CLINICAL IMPLICATIONS

Recent discoveries elucidating striatal pathway control of decision-making and behavior may also help to develop our understanding of the neuropathologies associated with dysfunction of the striatum.

### PARKINSON’S DISEASE

Bilateral optical excitation of striatal *indirect* pathway neurons results in a Parkinsonian state, inducing increased freezing, bradykinesia and decreased locomotor initiations (Kravitz et al., 2010). Conversely, in a mouse model of Parkinson’s disease, stimulation of *direct* pathway neurons rescued deficits in freezing, bradykinesia and initiation of ambulation (Kravitz et al., 2010). These data indicate that Parkinsonian symptoms may result from an overactivation of the *indirect* pathway, highlighting the efficacy of treatments that act to increase activity in the *direct* pathway and reduce activity in the *indirect* pathway.

### DRUG ABUSE

Optogenetic and gene-manipulation studies demonstrate activity within *direct* and *indirect* pathway NAc neurons to bidirectionally control both psychostimulant-induced locomotor sensitisation and CPP, facilitating or attenuating responses, respectively (Hikida et al., 2010; Lobo et al., 2010; Ferguson et al., 2011; Chandra et al., 2013). Similarly, optical activation of NAc D1-, but not D2-MSNs enhances morphine CPP (Koo et al., 2014). Indeed, recent evidence reveals expression of μ-opioid receptors within NAc D1-MSNs of the *direct* pathway to be necessary to support opiate-induced CPP and locomotor sensitization (Cui et al., 2014). These data are congruent with a model of striatal functioning proposing *direct* pathway neurons to control reinforcement learning, and *indirect* pathway neurons to mediate aversive learning and punishment (Hikida et al., 2010; Kravitz et al., 2012; Nakanishi et al., 2014). Interestingly, recent evidence has revealed that increased activity in the *indirect* pathway also promotes resilience to compulsive cocaine-seeking (Bock et al., 2013). It is hypothesized that this may be a relevant to the ability of *indirect* pathway NAc neurons to reduce perseveration during reward learning (Yawata et al., 2012; Nakanishi et al., 2014).

### OBESITY

A link between compulsive eating and altered striatopallidal transmission has also recently been revealed (Kenny et al., 2013). Compulsive-like food intake in obese rats is associated with a downregulation of dopamine D2 receptors within the striatum (Johnson and Kenny, 2010). Accordingly, viral knockdown of striatal D2 receptors accelerated the development of compulsive food-seeking behavior in rats (Johnson and Kenny, 2010). While yet to be investigated, the authors of this study hypothesize that activity within the *indirect* pathway may control compulsive food-seeking in the same way that it controls compulsive drug-seeking (Bock et al., 2013; Kenny et al., 2013).

### AUTISM SPECTRUM DISORDERS (ASDs)

Recent evidence has also indicated a link between striatal dysfunction and ASDs. Mutations of neuroligin-3, a postsynaptic cell-adhesion molecule that’s disruption is associated with ASDs, specifically impeded synaptic inhibition onto D1- but not D2-MSNs within the NAc in mice (Rothwell et al., 2014). The resulting disinhibition of NAc D1-MSNs was associated with enhanced acquisition repetitive motor behaviors, typical of ASDs, predicted to be resultant of facilitated signaling through the *direct* pathway.

### SCHIZOPHRENIA

It is still unclear how activity within output pathway neurons may contribute to other disorders associated with dysfunction of the striatum, including schizophrenia. However, evidence that two different transgenic mice lines demonstrating schizophrenia-like behavioral abnormalities show increased expression levels of D2-receptor RNA and protein within the striatum, suggests that altered activity in the *indirect* pathway may contribute to the etiology of schizophrenia (Jaaro-Peled et al., 2013; Niwa et al., 2013).

## CONCLUSION AND FUTURE DIRECTIONS

With recent advances in technologies allowing the specific investigation of striatal cell-types and output pathways, the role of the striatum in controlling economical decision-making has become increasingly clearer. Specifically, the majority of evidence indicates that the *direct* and *indirect* striatal pathways act in an opposing manner to control behavior (Lobo and Nestler, 2011; Nakanishi et al., 2014). In general, activation of D1-MSNs within the *direct* pathway promote actions that result rewarding outcomes, while activity within the indirect pathway is necessary to avoid punishment, as well as inhibit learnt behaviors, thus allowing behavioral flexibility (Hikida et al., 2010; Kravitz et al., 2012; Yawata et al., 2012). This has been hypothesized to be facilitated by dopamine induced modifications in the influence of limbic inputs into D1-MSNs and cortical inputs onto D2-MSNs, produced by the presentation or omission of rewarding and aversive stimuli (Grace et al., 2007; Nakanishi et al., 2014).

There is now a compelling body of evidence indicating that a decrease in dopamine D2 receptors within the striatum is associated with compulsive behaviors, likely through a loss of inhibitory control (Johnson and Kenny, 2010; Bock et al., 2013). Given the hypothesized role of cortical projections onto striatal D2-MSNs in controlling the ability to flexibly switch between behaviors, it could be predicted that optical activation cortical neurons may be effective in the treatment of maladaptive compulsive behaviors. Indeed, this may be especially true of D2-MSNs within the DLS, an area implicated in habit formation (Yin et al., 2004; Seger and Spiering, 2011).

Finally, it is important to note that the vast majority striatal manipulations described within this review involve the activation or inhibition of large populations of MSNs, however, NAc MSNs encoding separate action representations are thought to be contained within discrete ensembles of as little as 2% of total NAc MSNs (Nicola et al., 2004; Mattson et al., 2008). Future research should seek to further investigate the cellular make-up of these ensembles and elucidate how specific patterns and timing of D1- and D2-MSN activation may act to control economical decision-making.

## Conflict of Interest Statement

The authors declare that the research was conducted in the absence of any commercial or financial relationships that could be construed as a potential conflict of interest.
